# 

**Published:** 1911-07-22

**Authors:** 


### BOOKS RECEIVED.

W. B. Saunders Company.

"Hospital Management." By Charlotte A. Aikens.

Percy Lindley.

"Tourist Guide to the Continent." By the Great Eastern Railway Company.

### MEDICAL CHAIRS IN GLASGOW UNIVERSITY.

An ordinance of the University Court of the University of Glasgow relating to medical chairs awaits the approval of the Privy Council. In February of this year an agreement was entered into among the University Court of Glasgow, the Royal Infirmary of Glasgow, St. Mungo's College, Glasgow, and the trustees of the late Henry Muirhead, M.D., constituted by the Muirhead Order Confirmation Act, 1910, by which *inter alia* funds are provided for the foundation of certain chairs and for augmenting the emoluments of the existing chairs of clinical medicine and clinical surgery. The ordinance provides for a professorship of pathology, to be designated the St. Mungo (Notman) Chair of Pathology (emolument £600); a professorship of obstetrics and gynæcology, to be designated the Muirhead Chair of Obstetrics and Gynæcology (£500); these two chairs are in addition to the existing chairs of pathology and midwifery. The ordinance also provides that the designation of the present professorship of clinical medicine shall be altered to the Muirhead Professorship of Medicine (£500), and that the designation of the present professorship of clinical surgery shall be altered to the St. Mungo Professorship of Surgery (£500). The provisions of the ordinance will not affect any professor holding a professorship at the date of the approval of the ordinance unless the professor consents.

Miss Arabella Catherine Hankey, of Warwick Square, has bequeathed £500 for religious work at home, and " some hospital or hospitals (e.g. Grosvenor Hospital or Westminster Hospital)."

### MEDICAL APPOINTMENTS.

Dr. W. A. B. Young, M.R.C.S., has been elected second assistant medical officer at the Out-Patient Department of the Manchester Children's Hospital at Pendlebury.

Mr. A. H. Payan Dawnay, F.R.C.S., has been appointed ophthalmic surgeon to out-patients at the Great Northern Central Hospital, Holloway Road, N.

Dr. W. Odell, M.D., has been appointed honorary consulting physician to the Western Hospital, Torquay. Dr. J. E. Paul, M.D., has been appointed honorary physician, a post which Dr. Odell has resigned after holding it for sixteen years.

Dr. F. Craven Moore, M.D., M.Sc. (Manch.), F.R.C.P. (Lond.), has been appointed an honorary assistant physician, and Mr. H. H. Rayner, M.B., Ch.B. (Manch.), F.R.C.S. (Eng.), has been appointed an honorary assistant surgeon to the Manchester Royal Infirmary.

Mr. D. K. Coutts, M.B., B.S. Lond., F.R.C.S. Eng., has been elected third honorary assistant surgeon at the Norfolk and Norwich Hospital. Dr. H. J. Starling and Dr. Henry Watson have been elected honorary anæsthetists. The house physician, Dr. William A. Watson, and the house surgeon, Mr. W. L. Holyoak, have resigned, and Mr. J. P. Charles and Mr. R. F. P. Cory have been appointed to fill their vacancies. An additional resident medical officer is appointed to attend the Casualty Department.

The following appointments have been made by the Manchester University: Lecturer in Orthopædic Surgery, Mr. Charles Roberts, M.B., B.S. (London), F.R.C.S. Mr. Roberts graduated with Honours in Medicine at the University of London in 1896, and obtained the Diploma of Fellow of the Royal College of Surgeons in 1899. Assistant Lecturers in Surgery, Mr. P. R. Wrigley, F.R.C.S., and Mr. Garnett Wright, F.R.C.S. Mr. Wrigley, who in 1905 obtained the Diploma of Fellow of the Royal College of Surgeons, was a student of Owens College, and in 1900 he obtained the Bradley Memorial Scholarship in Clinical Surgery at the Manchester Royal Infirmary. Mr. Wright was awarded medals in Practical Anatomy and Pathology, and prizes in Surgery during his career as a student of Edinburgh University. In 1900 he graduated with Honours, and in 1905 obtained the Diploma of Fellow of the Royal College of Surgeons of England.

### APPOINTMENTS VACANT.

Full particulars of these vacancies can be obtained on reference to our advertisement columns:

Coventry and Warwickshire Hospital.—Junior House Surgeon.

Kensington and Fulham General Hospital.—Anæsthetist, Registrar.

Leicester Infirmary.—Assistant House Surgeon.

Prince of Wales's General Hospital.—Junior House Physician.

Sir John Morris, K.C.S.I., has furnished the King Edward VII. Memorial Ward in the new Sir Henry Tyler wing of the London Homœopathic Hospital, Great Ormond Street, W.C., as a memorial to the late Lady Morris.

The committee of the Church Missionary Society have recently agreed to the establishment of a hospital at Charsadda as a branch of the Peshawar Medical Mission. The fitting up of a serai for the purpose of a hospital will cost about £100. The work was started in April of this year, and about two hundred patients are seen daily.

